# Effects of eculizumab and rituximab on visual function, motor function and social quality in patients with NMOSD: a comparative study

**DOI:** 10.3389/fneur.2025.1698950

**Published:** 2025-11-05

**Authors:** Yihan Liu, Li Meng, Jiyuan Li, Xiao Wang, Xiaoman Zhang, Xiangzeng Kong, Yongzhi Zhang

**Affiliations:** ^1^Department of Neurology, The First Hospital of Hebei Medical University, Shijiazhuang, Hebei, China; ^2^Hebei Hospital of Xuanwu Hospital Capital Medical University, Shijiazhuang, Hebei, China; ^3^Brain Aging and Cognitive Neuroscience Laboratory of Hebei Province, Shijiazhuang, Hebei, China; ^4^Neuromedical Technology Innovation Center of Hebei Province, Shijiazhuang, Hebei, China; ^5^Department of Neurology, The First Hospital of Shanxi Medical University, Taiyuan, Shanxi, China

**Keywords:** eculizumab, rituximab, NMOSD, visual function, motor function, social quality

## Abstract

**Objective:**

To compare the effects of eculizumab and rituximab on visual function, motor function, and social quality of life in patients with aquaporin-4 antibody positive neuromyelitis optica spectrum disorder (NMOSD).

**Methods:**

This retrospective study included NMOSD patients treated with eculizumab (EG, *n* = 114) or rituximab (CG, *n* = 114) at the Department of Neurology, First Hospital of Hebei Medical University, and the First Hospital of Shanxi Medical University between January 2018 and August 2024. Patients were followed for 6 months to assess changes in visual, motor, and social outcomes.

**Results:**

Baseline characteristics, including demographics, disease duration, AQP4-IgG status, annual recurrence rate, EDSS score, and comorbidities, were comparable between groups (*P* > 0.05). Both groups showed gradual improvement in visual acuity after treatment (*P* < 0.05). However, the EG demonstrated significantly better uncorrected visual acuity at 1, 3, and 6 months (*P* < 0.001) and higher corrected visual acuity (*P* < 0.05). Visual field defects decreased more in the EG (61.40%−33.33%) compared with the CG (64.91%−50.88%, *P* < 0.05). Abnormal color vision improved in both groups, with greater reduction in the EG, though not statistically significant at 6 months (*P* = 0.065). Motor function outcomes showed significant time, group, and interaction effects (*P* < 0.001). The EG achieved higher Fugl-Meyer Assessment (FMA) scores and lower Modified Ashworth Scale (MAS) scores at all time points (*P* < 0.05). Social function (SFS) improved significantly in both groups, with greater gains in the EG (*P* < 0.001). Anxiety (HAMA) and depression (HAMD) scores decreased in both groups, but reductions were more pronounced in the EG (*P* < 0.001).

**Conclusion:**

NMOSD significantly impairs visual, motor, and social functions. Eculizumab demonstrated superior efficacy over rituximab in improving functional outcomes and quality of life, supporting its role as an effective therapeutic option for NMOSD.

## 1 Introduction

Neuromyelitis optica spectrum disorder (NMOSD) is a rare, relapsing autoimmune condition of the central nervous system, primarily affecting the optic nerves and spinal cord (1). It is strongly associated with autoantibodies against aquaporin-4 (AQP4-IgG), which serve as a diagnostic marker in approximately 70–80% of patients (2). The incidence and prevalence of NMOSD are estimated to be 0.05–0.40 per 100,000 and 0.52-4.4 per 100,000 individuals, respectively (3, 4), and at higher risk observed among Asian and African populations (5, 6).

Optic neuritis, resulting in visual impairment, is among the most common initial manifestations of AQP4-IgG positive NMOSD, along with transverse myelitis, with both symptoms often occurring in early disease stages (7). Approximately 50%−80% of NMOSD patients develop optic neuritis during the early stages of the disease, presenting with sudden loss of vision in one or both eyes, visual field constriction, or central scotoma (8). This visual impairment is frequently severe, recurrent, and progressive. Despite treatment, some patients experience only partial or limited visual recovery. Additionally, spinal cord lesions associated with NMOSD often result in motor dysfunction, typically characterized by limb weakness, muscle atrophy, and paresthesia (9). These motor impairments significantly compromise mobility, making daily activities such as dressing, eating, and navigating stairs difficult or impossible without assistance, thereby severely limiting patients' independence. NMOSD also profoundly affects patients' social quality of life. Visual and motor impairments often hinder participation in social activities, leading to withdrawal and isolation. Social isolation arises from an interplay of physiological, psychological, cognitive, and environmental factors, and its effects span physical health, emotional well-being, family dynamics, and broader social interactions (10). In addition, chronic pain and sustained functional limitations may contribute to psychological issues such as depression and anxiety, which in turn negatively impact social relationships and overall life satisfaction. These psychosocial challenges not only increase the disease burden but may also hinder rehabilitation and long-term recovery.

Over the last decade, major progress has been made in understanding the immunopathology of NMOSD and in developing disease-modifying therapies (DMTs) such as rituximab and eculizumab. These therapies have demonstrated efficacy in reducing relapse rates, which is a critical endpoint in clinical trials. However, far less is known about their impact on persistent symptoms and functional impairments that affect patients' quality of life, including visual deficits, motor dysfunction, pain, fatigue, and psychosocial consequences.

Current NMOSD treatment strategies largely focus on immunosuppression and relapse prevention. However, many patients continue to experience debilitating symptoms between relapses. These symptoms, such as poor visual recovery after optic neuritis, persistent spasticity, and reduced social participation, are major determinants of long-term disability. Despite their prevalence, symptom control outcomes have not been adequately explored in NMOSD trials.

Eculizumab, a complement C5 inhibitor, is approved specifically for AQP4-IgG positive NMOSD. It blocks the terminal complement cascade and may exert neuroprotective effects that extend beyond relapse suppression. Rituximab, though used off-label, remains a common first-line immunotherapy. Recent real-world data and retrospective analyses have begun to explore head-to-head comparisons of these therapies (11, 12), yet few studies have comprehensively evaluated their differential effects on functional recovery, particularly in domains such as vision, motor ability, and social function. Whether these agents differ in their ability to improve symptomatic outcomes is still unclear.

Therefore, this study aims to conduct a comparative evaluation of the therapeutic effectiveness of eculizumab and rituximab in patients with AQP4-IgG–positive NMOSD, focusing on improvements in visual function, motor function, and quality of life over a 6-month follow-up period.

## 2 Materials and methods

### 2.1 General information

**Figure 1:**
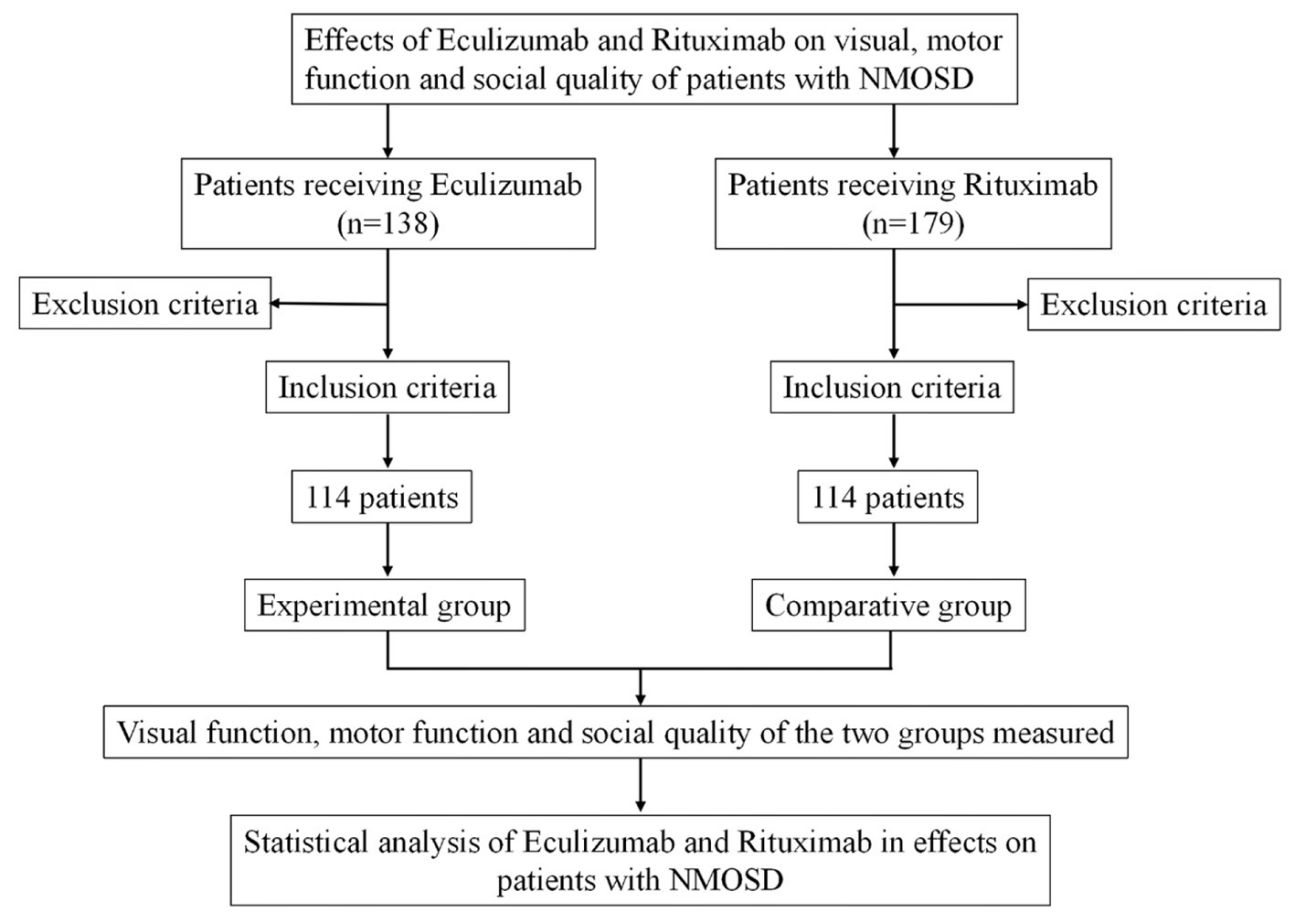
Study cohort screening.

A retrospective analysis was carried out on clinical data from patients diagnosed with NMOSD who received either eculizumab or rituximab at the Department of Neurology, the First Hospital of Hebei Medical University and the First Hospital of Shanxi Medical University between January 2018 and August 2024. Based on the treatment regimen, patients were divided into two groups: the experimental group (EG), which received eculizumab (*n* = 114), and the comparative group (CG), which received rituximab (*n* = 114). All patients were followed for a period of 6 months. The study was approved by the Ethics Committee of the First Hospital of Hebei Medical University (approval No. HFH202505161). Given the retrospective nature of the study and the use of anonymized data, the requirement for written informed consent was waived. A flow diagram illustrating the cohort selection process is presented in Figure 1.

Inclusion criteria: (1) Diagnosis of NMOSD based on the 2015 revised international consensus diagnostic criteria (11), confirmed through imaging and laboratory findings (13). (2) Age between 18 and 65 years. (3) Availability of follow-up records for at least 6 months after diagnosis. (4) Availability of anonymized clinical and follow-up records; written informed consent was waived by the Ethics Committee due to the retrospective design. (5) Confirmed AQP4-IgG seropositivity by a cell-based assay.

Exclusion criteria: (1) Presence of other major neurological disorder, such as multiple sclerosis or stroke. (2) Severe mental illness or cognitive impairment. (3) Diagnosis of malignant diseases, including any type of tumors. (4) History of major surgery or trauma within the past year. 5) Current pregnancy or breastfeeding. 6) Presence of other conditions that may affect the optic nerve.

### 2.2 Data collection

(1) Visual Function Assessment.

1) Visual Acuity Testing: Uncorrected and best-corrected visual acuity were measured using a standard eye chart under controlled lighting conditions.
2) Visual Field Assessment: Visual field defects were evaluated using a perimeter to assess the extent and pattern of visual field loss.
3) Color Vision Testing: Color vision abnormalities were assessed using a standardized color vision test.

(2) Motor Function Assessment.

1) Muscle Tension Assessment: Muscle tone was assessed using the Modified Ashworth Scale (MAS) (14). Examiners passively moved the patient's limbs to evaluate resistance levels, with higher scores indicating increased muscle spasticity.
2) Motor Function Assessment: The Fugl-Meyer Motor Function Assessment Scale (FMA) was used to evaluate motor function in both upper and lower extremities. The FMA is a validated tool for assessing motor recovery, covering domains such as movement, reflex activity, balance, joint motion, and pain. Scoring was based on the examiner's observation of specific motor tasks (e.g., arm raising, finger dexterity, leg extension), with higher scores reflecting better motor performance.

(3) Social quality assessment.

1) Social Functioning Scale (SFS): The SFS was employed to assess social participation and functioning. This scale evaluates the frequency of social interactions, quality of relationships, level of social support, and overall engagement in social activities (15).
2) Hamilton Anxiety Rating Scale (HAMA): Anxiety symptoms were measured using the HAMA, which assesses psychological and somatic aspects of anxiety, including emotional tension, sleep disturbances, and autonomic symptoms.
3) Hamilton Depression Rating Scale (HAMD): Depressive symptoms were assessed using the HAMD, focusing on domains such as mood, interest levels, sleep quality, psychomotor retardation, and self-blame.

### 2.3 Therapeutic method

#### 2.3.1 Experimental group

The treatment duration for the experimental group was six months. Patients in this group received eculizumab according to the following protocol.

Pre-treatment Preparation: To minimize the risk of infusion-related reactions, all patients received 20 mg of intramuscular diphenhydramine and 1000 mg of oral acetaminophen 30 minutes prior to each eculizumab infusion.

Induction Phase (Weeks 1–4): Eculizumab was administered at a dose of 900 mg via intravenous infusion once weekly for four consecutive weeks.

Maintenance Phase (From Week 5 onward): Starting in the fifth week, the dosage was adjusted to 1,200 mg of eculizumab via intravenous infusion every two weeks.

#### 2.3.2 Comparative group

The comparative group was treated with rituximab as follow:

Pre-treatment Preparation: Thirty minutes before rituximab administration, patients were given 20 mg of diphenhydramine intramuscularly and 300 mg of oral ibuprofen to prevent potential infusion-related reactions.

Dosing Regimen: Rituximab was administered intravenously at a dosage of 1000 mg on day 1 and 1,000 mg on day 15 as the induction phase. Maintenance therapy consisted of 500 mg every 6 months, with dosing intervals adjusted based on CD19 or CD20 B-cell monitoring and relapse status. This regimen aligns with widely used protocols for NMOSD treatment.

Maintenance and Duration: After the initial four-week induction phase, rituximab was administered as needed based on clinical evaluation, with a total treatment duration of six months.

#### 2.3.3 Timing of therapy initiation and relapse management

All included patients experienced a recent clinical relapse prior to the initiation of immunotherapy. Acute relapse episodes were treated with intravenous methylprednisolone (1 g/day for 3–5 consecutive days). For patients who exhibited suboptimal response to steroids, therapeutic plasma exchange (PLEX) was administered (5–7 sessions).

Following clinical stabilization, rituximab or eculizumab treatment was initiated between 14 and 30 days after the completion of relapse therapy, depending on the patient's recovery and safety evaluation. This standardized timing ensured that the effects measured in the study reflected the maintenance and symptomatic impact of the therapies rather than acute relapse treatment effects.

### 2.4 Statistical analysis

All statistical analyses were performed using SPSS version 22.0. Data with a normal distribution are presented as mean ± standard deviation (*x* ± *s*), and comparisons between groups were conducted using the independent samples *t*-test. For non-normally distributed data, results are presented as median [interquartile range, IQR], and group comparisons were analyzed using the Mann–Whitney U-test. Categorical variables are expressed as frequencies (*n*) and percentages (%), and comparisons were made using the chi-square test or Fisher's exact test, as appropriate. Repeated measures ANOVA was employed to assess changes over time within and between groups. When significant differences were found, Bonferroni correction was used for *post hoc* comparisons. The Shapiro–Wilk test was used to test for normality. A two-tailed *P*-value <0.05 was considered statistically significant. Missing data were excluded listwise and were minimal (<5%), thus not imputed.

## 3 Results

### 3.1 General baseline information

There was no statistical difference involving the EG and the CG in terms of age, gender, BMI, course of disease, EDSS score, AQP4-IgG positivity, annual recurrence rate, hypertension, and diabetes (*P* > 0.05, Table 1), indicating that the two groups were well comparable. The median EDSS score of both groups was 3.5, indicating that the patients were at a moderate disability level overall. Additionally, all patients included in the final analysis were AQP4-IgG positive, in accordance with the approved indication of eculizumab.

**Table 1:** General baseline information.

**Characteristics**	**Experimental group (*n =* 114)**	**Comparative group (*n =* 114)**	***t*/*U*/χ^2^**	***P*-value**
Age (years, mean ± SD)	42.7 ± 12.3	43.5 ± 11.8	*t =* −0.51	0.610
Gender [female, *n* (%)]	92 (80.7%)	88 (77.2%)	χ^2^ = 0.44	0.507
BMI [(kg/m^2^), mean ± SD]	23.5 ± 3.2	24.1 ± 3.5	*t* = −1.36	0.175
Disease duration [months, median (IQR)]	36 (18–60)	39 (20-65)	*Z* = −0.76	0.449
EDSS [score, median (IQR)]	3.5 (2.0–5.0)	3.5 (2.5-5.5)	Z = −0.52	0.601
AQP4-IgG [positive, *n* (%)]	99 (86.8%)	96 (84.2%)	χ^2^ = 0.33	0.567
Annual relapse [rate, (mean ± SD)]	1.2 ± 0.8	1.3 ± 0.9	*t =* −0.89	0.372
Hypertension	15 (13.2%)	18 (15.8%)	χ^2^ = 0.33	0.567
Diabetes	9 (7.9%)	7 (6.1%)	χ^2^ = 0.27	0.602

EDSS, Extended Disability Status Scale; AQP4-IgG, aquaporin 4 antibody.

### 3.2 Dynamic changes of visual function within 6 months of follow-up after treatment

Notable differences were observed between the comparative group (CG) and experimental group (EG) in terms of uncorrected visual acuity, corrected visual acuity, and visual function outcomes, with significant effects noted over time, between groups, and in time × group interactions.

#### 3.2.1 Uncorrected visual acuity

Repeated measures ANOVA showed significant main effects of time *F*_(time)_ = 185.23, *P* < 0.001, group *F*_(group)_ = 12.76, *P* < 0.001, and time × group interaction *F*_(interaction)_ = 9.453, *P* < 0.001. Uncorrected visual acuity improved significantly over time in both groups. At 1 month after treatment, acuity was significantly higher than baseline; at 3 months after treatment, it surpassed both BT and 1-month levels; and at 6 months after treatment, it was significantly higher than all previous time points (*P* < 0.05). At each post-treatment time point, the EG demonstrated significantly better uncorrected visual acuity compared to the CG:1 month after treatment: *t* = 4.66, *P* < 0.001; 3 months after treatment: *t* = 6.53, *P* < 0.001; 6 months after treatment: *t* = 8.00, *P* < 0.001.

#### 3.2.2 Corrected visual acuity

Corrected visual acuity also improved significantly over time *F*_(time)_ = 132.58, *P* < 0.001, with significant group *F*_(group)_ = 8.92, *P* = 0.003, and interaction effects *F*_(interaction)_ = 5.73, *P* = 0.012. At each follow-up point (1, 3, and 6 months), corrected visual acuity in both groups was significantly higher than baseline. Additionally, the EG exhibited superior corrected acuity compared to the CG: 1 month after treatment: *t* = 2.45, *P* = 0.015; 3 months after treatment: *t* = 3.97, *P* < 0.001; 6 months after treatment: *t* = 3.68, *P* < 0.001 (Table 2).

**Table 2:** Dynamic changes of uncorrected and best corrected visual acuity over time (Mean ± SD).

**Visual parameter**	**Group (*n =* 114)**	**Before treatment**	**1 Month after treatment**	**3 Month after treatment**	**6 Month after treatment**	**t**	***P* value**
Uncorrected visual acuity	Comparative group	0.33 ± 0.14	0.42 ± 0.13^a^	0.53 ± 0.18^ab^	0.56 ± 0.16^abc^	8.00	<0.001
	Experimental group	0.34 ± 0.13	0.51 ± 0.16^a^	0.69 ± 0.19^ab^	0.77 ± 0.23^abc^		
Best corrected visual	Comparative group	0.52 ± 0.17	0.60 ± 0.19^a^	0.64 ± 0.18^ab^	0.68 ± 0.20^abc^	3.68	<0.001
	Experimental group	0.51 ± 0.19	0.66 ± 0.18^a^	0.74 ± 0.20^ab^	0.78 ± 0.21^abc^		

^a^Compared with BT, *P* < 0.05; ^b^Compared with 1 month AT, *P* < 0.05; ^c^Compared with 3 months AT, *P* < 0.05.

#### 3.2.3 Visual field defects

**Figure 2:**
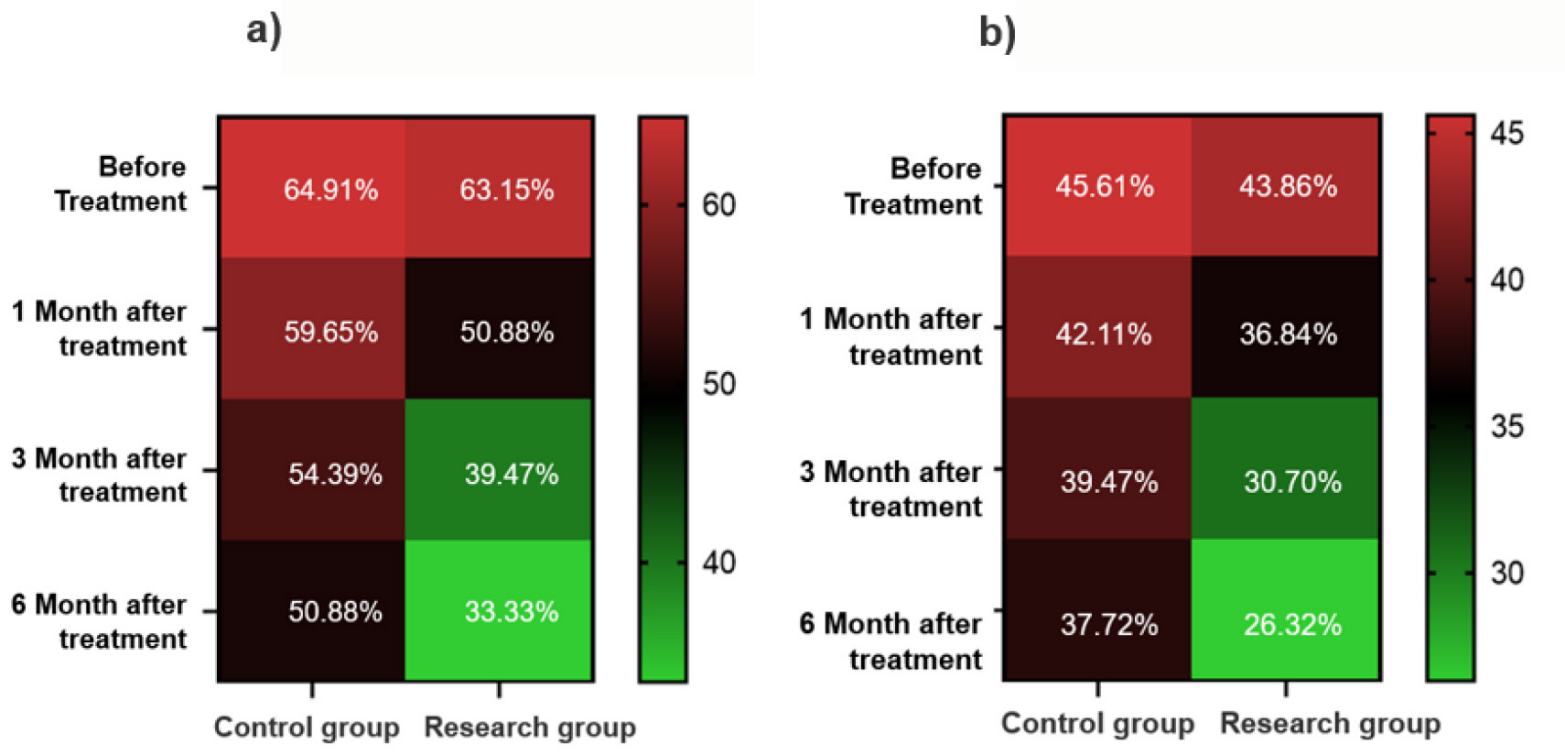
Heat map of visual function recovery. **(a)** Changes in the proportion of patients with visual field defects. **(b)** Changes in the proportion of patients with color vision abnormalities.

Both groups showed improvements in visual field defects over time, though the degree and rate of improvement differed substantially. In the EG, the proportion of patients with visual field defects decreased from 61.40% at baseline to 33.33% at 6 months, an improvement of 28.07%. Significant improvement was observed from 3 months onward (*P* < 0.05), with the most pronounced effects at 6 months (Table 3; Figure 2a). In contrast, the CG exhibited a smaller reduction in visual field defects, from 64.91% at baseline to 50.88% at 6 months, reflecting only a 14.03% improvement. Between-group comparisons revealed significantly lower rates of visual field defects in the EG compared to the CG at: 3 months: *P* = 0.024; 6 months: *P* = 0.007.

**Table 3:** Dynamic changes of visual field defects and color vision abnormalities in the two groups of patients during the 6-month follow-up.

**Visual function**	**Group (*n =* 114)**	**Before treatment**	**1 Month after treatment**	**3 Month after treatment**	**6 Month after treatment**	**χ^2^**	***P*-value**
Visual field defects [*n* (%)]	Comparative group	74 (64.91%)	68 (59.65%)	62 (54.39%)	58 (50.88%)^a^	2.683	0.007
	Experimental group	70 (61.40%)	58 (50.88%)	45 (39.47%)^a^	38 (33.33%)^ab^		
Color vision abnormalities [*n* (%)]	Comparative group	52 (45.61%)	48 (42.11%)	45 (39.47%)	43 (37.72%)	1.845	0.065
	Experimental group (*n =* 114)	50 (43.86%)	42 (36.84%)	35 (30.70%)^a^	30 (26.32%)^a^		

^a^Compared with before treatment, *P* < 0.05; ^b^Compared with 1 month after treatment, *P* < 0.05; ^c^Compared with 3 months after treatment, *P* < 0.05.

#### 3.2.4 Color vision abnormalities

Improvements in color vision were less pronounced than those in visual field function, though the EG still demonstrated a clear advantage. The proportion of patients with abnormal color vision in the EG decreased from 43.86% at baseline to 26.32% at 6 months, with statistically significant improvements observed at both 3 and 6 months (*P* < 0.05). In the CG, the reduction was more modest, from 45.61% to 37.72%, and did not reach statistical significance. While the between-group difference at 6 months did not achieve significance, the proportion of abnormal color vision in the EG was 11.40% lower than in the CG, approaching statistical significance (*P* = 0.065) (Table 3; Figure 2b).

### 3.3 Dynamic changes in motor function over 6 months of follow-up

Significant changes were observed in both MAS and FMA scores over time, between groups, and in their interaction effects: MAS: *F*_(time)_ = 105.62, *P* < 0.001; *F*_(group)_ = 22.46, *P* < 0.001; *F*_(interaction)_ = 78.45, *P* < 0.001; FMA: *F*_(time)_ = 198.74, *P* < 0.001; *F*_(group)_ = 11.24, *P* < 0.001; *F*_(interaction)_ = 62.83, *P* < 0.001.

#### 3.3.1 MAS scores

MAS scores decreased significantly in both groups at 1 month, 3 months, and 6 months AT compared to baseline (*P* < 0.05). Moreover, the EG consistently exhibited lower MAS scores than the CG at all follow-up time points, indicating a greater reduction in muscle spasticity: 1 month AT: *t* = 2.46, *P* = 0.014; 3 months AT: *t* = 6.87, *P* < 0.001; 6 months AT: *t* = 10.14, *P* < 0.001 (Table 4).

**Table 4:** Dynamic changes in motor function of the two groups of patients within 6 months of follow-up after treatment.

**Variables**	**Groups (*n =* 114)**	**Before treatment**	**1 Month after treatment**	**3 Month after treatment**	**6 Month after treatment**	***t***	***P*-value**
MAS	Comparative group	2.57 ± 0.93	2.12 ± 0.85^a^	1.88 ± 0.53^ab^	1.45 ± 0.39^abc^	10.14	<0.001
	Experimental group	2.48 ± 0.86	1.89 ± 0.45^a^	1.46 ± 0.38^ab^	1.03 ± 0.17^abc^		
FMA	Comparative group	126.88 ± 20.45	145.62 ± 22.37^a^	165.73 ± 24.81^ab^	180.95 ± 26.54^abc^	−5.54	<0.001
	Experimental group	128.43 ± 18.59	168.75 ± 21.92^a^	185.16 ± 22.05^ab^	201.38 ± 29.17^abc^		

^a^Compared with before treatment, *P* < 0.05; ^b^Compared with 1 month after treatment, *P* < 0.05; ^c^Compared with 3 months after treatment, *P* < 0.05.

#### 3.3.2 Motor function (FMA scores)

FMA scores significantly increased in both groups at each follow-up point compared to baseline (*P* < 0.05), indicating improved motor function. The EG demonstrated significantly higher FMA scores than the CG at each time point: 1 month after treatment: *t* = −7.67, *P* < 0.001; 3 months after treatment: *t* = −6.34, *P* < 0.001; 6 months after treatment: *t* = −5.54, *P* < 0.001 (Table 4).

These findings suggest that patients in the experimental group experienced more substantial improvements in muscle tone and motor function over the 6-month follow-up period.

### 3.4 Dynamic changes in social function within 6 months of follow-up

Significant differences were observed in SFS, HAMA, and HAMD scores over time, between groups, and in interaction effects: SFS: *F*_(time)_ = 84.23, *P* < 0.001; *F*_(group)_ = 37.46, *P* < 0.001; *F*_(interaction)_ = 62.31, *P* < 0.001. HAMA: *F*_(time)_ = 103.55, *P* < 0.001; *F*_(group)_ = 47.52, *P* < 0.001; *F*_(interaction)_ = 54.70, *P* < 0.001. HAMD: *F*_(time)_ = 133.12, *P* < 0.001; *F*_(group)_ = 26.85, *P* < 0.001; *F*_(interaction)_ = 89.35, *P* < 0.001

#### 3.4.1 Social function (SFS scores)

SFS scores significantly increased in both groups at 1 month, 3 months, and 6 months after treatment compared to baseline (*P* < 0.05). At each time point, the EG demonstrated significantly greater improvement in social functioning than the CG:1 month after treatment: *t* = 5.757, *P* < 0.001; 3 months after treatment: *t* = 10.14, *P* < 0.001; 6 months after treatment: *t* = 12.26, *P* < 0.001 (Table 5).

**Table 5:** Dynamic changes in social function of the two groups of patients within 6 months of follow-up.

**Variables**	**Groups (*n =* 114)**	**Before treatment**	**1 Month after treatment**	**3 Month after treatment**	**6 Month after treatment**	***t***	***P*-value**
SFS-36	Comparative group	42.17 ± 9.32	49.51 ± 8.78^a^	53.33 ± 8.46^ab^	57.15 ± 9.35^abc^	−4.62	<0.001
	Experimental group	41.53 ± 8.46	54.66 ± 10.32^a^	59.38 ± 10.12^ab^	63.25 ± 10.31^abc^		
HAMA	Comparative group	22.55 ± 3.57	19.24 ± 2.58^a^	16.35 ± 2.88^ab^	14.22 ± 1.98^abc^	12.26	<0.001
	Experimental group	21.94 ± 3.07	17.13 ± 2.94^a^	13.22 ± 1.55^ab^	11.46 ± 1.35^abc^		
HAMD	Comparative group	24.58 ± 2.95	21.35 ± 2.34^a^	18.35 ± 1.62^ab^	16.62 ± 1.51^abc^	16.29	<0.001
	Experimental group	24.07 ± 3.14	18.35 ± 1.97^a^	14.26 ± 1.31^ab^	13.53 ± 1.38^abc^		

^a^Compared with before treatment, *P* < 0.05; ^b^Compared with 1 month after treatment, *P* < 0.05; ^c^Compared with 3 months after treatment, *P* < 0.05.

#### 3.4.2 Anxiety (HAMA scores)

HAMA scores decreased significantly over time in both groups, with scores at 1, 3, and 6 months AT all significantly lower than at baseline (*P* < 0.05). The EG showed significantly greater reductions in anxiety symptoms at each follow-up point: 1 month after treatment: *t* = 5.76, *P* < 0.001; 3 months after treatment: *t* = 10.14, *P* < 0.001; 6 months after treatment: *t* = 12.26, *P* < 0.001 (Table 5). Importantly, the magnitude of improvement in the EG exceeded the minimal clinically important difference (MCID) for HAMA, which is estimated at 2–3 points, indicating a clinically meaningful reduction in anxiety symptoms.

#### 3.4.3 Depression (HAMD scores)

In both groups, HAMD scores significantly declined at all follow-up points relative to baseline (*P* < 0.05). Specifically: 1 month after treatment: lower than baseline; 3 months after treatment: lower than baseline and 1 month after treatment; 6 months after treatment: lower than baseline, 1 month after treatment, and 3 months after treatment. At each follow-up, the EG demonstrated significantly lower HAMD scores compared to the CG, indicating greater improvement in depressive symptoms: 1 month after treatment: *t* = 10.01, *P* < 0.001; 3 months after treatment: *t* = 20.06, *P* < 0.001; 6 months after treatment: *t* = 16.29, *P* < 0.001 (Table 5). These improvements exceeded the MCID for HAMD (typically 4–5 points), indicating that the observed reductions were not only statistically significant but also clinically meaningful.

## 4 Discussion

NMOSD is an idiopathic inflammatory central nervous system disease characterized by the presence of AQP4 antibodies. Studies have found that the disease seriously affects patients' quality of life (16–18) and is closely related to depression and fatigue (18, 19). Given that eculizumab is indicated only for AQP4-IgG positive NMOSD, our study focused exclusively on this patient subgroup. This study evaluated the effects of rituximab and eculizumab on patients' visual function, motor function and social quality through a 6-month clinical intervention in patients with NMOSD. At 6 months, the intervention with eculizumab demonstrated moderate-to-large effect sizes across all domains, with particularly strong improvements in uncorrected visual acuity, muscle tone (MAS), depression (HAMD), and social functioning (SFS), suggesting a robust and clinically meaningful advantage over rituximab (Supplementary Table S1). The results show that eculizumab has achieved significant results in many aspects, but there are still some areas that need further exploration.

Optic neuritis is the primary manifestation induced by NMOSD, characterized by the concurrent involvement of ON and longitudinally extensive transverse myelitis (20). In the present study, NMOSD patients demonstrated significantly poorer uncorrected and corrected visual acuity compared to healthy controls. Furthermore, the prevalence of visual field defects and color vision abnormalities was notably elevated in this patient population. The severity of visual impairment varied, commonly reported as blurred vision or “inability to see clearly.” Symptom onset typically occurs within hours to a few days, with maximal visual loss developing over a two-week period. Although most patients retain a visual acuity above 0.1, approximately one-third may suffer more severe visual deficits. Visual recovery generally begins within several weeks and may continue over the course of up to a year. By the end of this recovery period, about 90% of patients achieve visual acuity levels of 0.5 or greater (21). Eculizumab exerts its therapeutic effect by binding to complement protein C5, thereby inhibiting its cleavage into C5a and C5b and ultimately preventing the formation of the membrane attack complex (MAC) (22). This blockade of the terminal complement cascade plays a pivotal role in attenuating the complement-mediated astrocyte damage characteristic of AQP4-IgG–positive NMOSD (23, 24). By contrast, rituximab targets CD20-expressing B cells, leading to their depletion and reducing the production of pathogenic AQP4 autoantibodies (25). However, B-cell depletion does not immediately impact preformed antibodies or complement activation. As a result, rituximab may be less effective in acutely controlling inflammatory damage, particularly during early stages of treatment. The more rapid and pronounced improvements in visual, motor, and psychosocial outcomes observed in the eculizumab group may therefore be attributed to its direct modulation of complement activity, allowing earlier interruption of the inflammatory cascade and enhanced tissue protection.

The various types of color vision disorders, such as red green and blue-yellow defects, have been identified in patients with optic neuritis. Typically, blue-yellow deficits are more prominent during the acute phase, whereas red-green defects are more frequently observed during the non-acute or recovery phase of the disease (26). In patients with NMOSD, approximately half experience visual field disturbances. These disturbances often present as central scotomas, vertical or arcuate defects, nasal field loss, quadrantanopia, and in severe cases, hemianopia or complete amaurosis. Some of these patterns are believed to result from involvement of the posterior optic nerve pathway (27).

Visual function tends to evolve during optic neuritis (ON), and pharmacological interventions can significantly influence this recovery trajectory. In the present study, treatment led to substantial improvements in both uncorrected and corrected visual acuity, indicating effective visual restoration. Notably, patients in the eculizumab group showed significantly greater and more rapid improvement in visual field defects compared to those treated with rituximab. This suggests that eculizumab may offer superior protection and recovery of optic nerve function, potentially due to its complement inhibition mechanism, which interrupts key inflammatory pathways involved in NMOSD pathogenesis. While improvements in color vision abnormalities did not reach statistical significance, the eculizumab group demonstrated an 11.40% lower incidence of color vision abnormalities at 6 months post-treatment compared to the rituximab group (*P* = 0.065), approaching significance. This finding suggests a potential benefit of eculizumab in supporting color vision recovery, which may become more apparent with a longer follow-up period or larger sample size (13). It is important to note that recovery of color vision and visual field function typically occurs more gradually and may require extended monitoring to fully capture the long-term therapeutic effects.

Unlike visual acuity, color vision involves more complex neural pathways, including the optic nerve, optic tract, visual radiation, and various structures within the visual cortex. Damage to these structures during the acute phase can lead to a prolonged recovery process, which may span months or even years, and in some cases, may not result in complete recovery (13). Therefore, the improvement of these functions may not be obvious in the short term after intervention. The results suggest that targeted treatment of color vision should be strengthened in future intervention programs. In visual outcomes, the eculizumab group showed significantly higher improvements in both uncorrected and corrected visual acuity at all follow-up points. Notably, the proportion of patients with visual field defects decreased by 28.1% in the eculizumab group vs. only 14.0% in the rituximab group. This suggests a more robust effect on optic nerve recovery, potentially due to the direct complement inhibition mechanism of eculizumab, which may reduce ongoing immune-mediated damage even after relapse resolution. Improvements in color vision abnormalities, while not statistically significant, also trended more favorably in the eculizumab group.

In terms of motor function, patients with NMOSD demonstrated significantly poorer performance on both the MAS and the FMA compared to healthy controls prior to intervention. NMOSD predominantly affects the optic nerve and spinal cord, which are critical components of the central nervous system. Spinal cord involvement disrupts the transmission of neural signals between the brain and peripheral limbs, leading to abnormal muscle control, increased muscle tone (spasticity), reduced motor function, and impaired balance. Moreover, the relapsing-remitting nature of NMOSD results in cumulative neurological damage over time. Even during periods of clinical stability, patients may experience persistent motor deficits, characterized by elevated muscle tone, diminished motor coordination, and compromised postural control (13). Following pharmacological intervention, patients exhibited a significant reduction in muscle spasticity and a marked improvement in motor function, indicating that treatment had a meaningful therapeutic effect on motor performance in NMOSD. However, many patients tend to reduce physical activity due to motor limitations, which can lead to disuse atrophy and further deterioration of functional capacity. Therefore, in clinical practice, it is essential to complement medical therapy with individualized physical rehabilitation programs and structured activity guidance. Such interventions can help mitigate secondary deconditioning, promote neuromuscular recovery, and improve long-term outcomes in NMOSD patients. In terms of motor recovery, patients treated with eculizumab achieved greater reductions in MAS scores, indicating better spasticity control, and higher FMA scores, reflecting improved limb coordination and strength. These findings are clinically meaningful, given the impact of motor deficits on daily independence.

NMOSD is a chronic and recurrent nervous system disease, and patients face many health problems such as vision loss and motor dysfunction for a long time. These physical discomforts and functional limitations will cause patients to feel inconvenient and embarrassed in social activities, thus reducing social participation. This behavior of reducing social activities may further lead to social isolation and increase the risk of anxiety and depression (28).

The social function evaluation results indicate that patients with NMOSD in this study sample have significantly poorer social participation, higher anxiety, and greater depression compared to healthy individuals. Following treatment with eculizumab, patients demonstrated significant improvements in social functioning, with notable reductions in anxiety and depression. These findings suggest that eculizumab not only benefits physiological function but also markedly enhances psychological well-being and social quality for NMOSD patients. Social and psychological outcomes followed similar trends. The SFS scores improved more in the eculizumab group, as did anxiety (HAMA) and depression (HAMD) scores. These improvements may result not only from better neurological recovery but also from reduced fear of relapse, improved physical function, and enhanced engagement in daily life. These findings align with prior evidence that eculizumab reduces relapse frequency more effectively than rituximab in AQP4+ NMOSD. However, our study extends this knowledge by showing that eculizumab may also yield superior post-relapse symptomatic recovery, a dimension not fully addressed in many clinical trials.

This study has several limitations that must be acknowledged. First, the retrospective design introduces inherent risks of selection bias, as treatment allocation was not randomized but based on clinical judgment, availability, or patient preference. Although baseline characteristics were statistically balanced between groups, unmeasured confounding factors (such as prior treatment history, relapse severity, or comorbid conditions) may still have influenced outcomes. Second, while our findings indicate that eculizumab led to significantly better improvements in visual acuity, motor function, and psychosocial outcomes compared to rituximab over a 6-month period, this timeframe represents a short- to medium-term observation window. Symptom control in NMOSD may evolve over longer periods due to factors such as neuroplasticity, recurrent disease activity, or delayed treatment effects. Therefore, although our data suggest early superiority of eculizumab, longer follow-up studies (e.g., 12–24 months) are warranted to determine the sustained impact of treatment on symptom progression, disability accumulation, and quality of life. Our findings should thus be interpreted as initial comparative evidence, not definitive long-term superiority. Third, the study lacked blinding, which may introduce observer or measurement bias, particularly in subjective outcome assessments such as visual acuity or social functioning. Lastly, although all patients received treatment after stabilization from an acute relapse, variability in the time to treatment initiation or prior relapse management (steroids vs. plasma exchange) could also influence recovery trajectories. Despite these limitations, the study provides valuable comparative data from a relatively large real-world cohort of AQP4-IgG positive NMOSD patients. Future prospective, randomized, and multicenter trials with longer follow-up are essential to validate and extend these findings.

## 5 Conclusion

Through a systematic evaluation of NMOSD patients, this study has demonstrated that the disease substantially impacts visual function, motor function, and social quality. The intervention with eculizumab has shown significant improvements in various aspects, including uncorrected visual acuity, corrected vision, visual field defects, muscle tension, motor ability, and alleviation of anxiety and depression symptoms. Although improvements in some indicators, such as abnormal color vision, did not achieve statistical significance, the study confirms the effectiveness of eculizumab in the clinical management of AQP4-IgG positive NMOSD and provides a solid foundation for further refining treatment strategies. Future research should focus on exploring extended and more targeted interventions to enhance the quality of life and functional recovery for NMOSD patients.

## Data Availability

The raw data supporting the conclusions of this article will be made available by the authors, without undue reservation.
