# Supplementary material for: Effects of eculizumab and rituximab on visual function, motor function and social quality in patients with NMOSD: a comparative study
Source: Front Neurol. 2025 Nov 5;16:1698950. doi: 10.3389/fneur.2025.1698950 (PMC12627026; doi:10.3389/fneur.2025.1698950)
Supplement: Supplementary file 1 [file Table_1.docx]

**Supplementary Table S1**

| Domain | Cohen’s d (EG vs CG at 6 months) | Interpretation |
| --- | --- | --- |
| Uncorrected Visual Acuity | 0.91 | Large |
| Corrected Visual Acuity | 0.52 | Moderate |
| MAS Score | 0.88 | Large |
| FMA Score | 0.67 | Moderate |
| HAMA Score | 0.76 | Moderate-to-Large |
| HAMD Score | 0.81 | Large |
| SFS Score | 0.79 | Large |
